# Reconstruction of a Comprehensive Interactome and Experimental Data Analysis of FRA10AC1 May Provide Insights into Its Biological Role in Health and Disease

**DOI:** 10.3390/genes14030568

**Published:** 2023-02-24

**Authors:** Theologia Sarafidou, Eleni Galliopoulou, Despina Apostolopoulou, Georgios A. Fragkiadakis, Nicholas K. Moschonas

**Affiliations:** 1 Department of Biochemistry and Biotechnology, University of Thessaly, Viopolis, 41500 Larissa, Greece; 2 Department of Biology, University of Crete, 71500 Heraklion, Greece; 3 Department of Nutrition and Dietetics Sciences, Hellenic Mediterranean University, Tripitos, 72300 Siteia, Greece; 4 School of Medicine, University of Patras, 26500 Patras, Greece; 5 Institute of Chemical Engineering Sciences, Foundation for Research and Technology Hellas (FORTH/ICE-HT), 26504 Patras, Greece

**Keywords:** spliceosome, expression profile, protein interactome, disease-protein network, RNA in situ hybridization, protein interactions methods

## Abstract

*FRA10AC1*, the causative gene for the manifestation of the *FRA10A* fragile site, encodes a well-conserved nuclear protein characterized as a non-core spliceosomal component. Pre-mRNA splicing perturbations have been linked with neurodevelopmental diseases. *FRA10AC1* variants have been, recently, causally linked with severe neuropathological and growth retardation phenotypes. To further elucidate the participation of FRA10AC1 in spliceosomal multiprotein complexes and its involvement in neurological phenotypes related to splicing, we exploited protein–protein interaction experimental data and explored network information and information deduced from transcriptomics. We confirmed the direct interaction of FRA10AC1with ESS2, a non-core spliceosomal protein, mapped their interacting domains, and documented their tissue co-localization and physical interaction at the level of intracellular protein stoichiometries. Although FRA10AC1 and SF3B2, a major core spliceosomal protein, were shown to interact under in vitro conditions, the endogenous proteins failed to co-immunoprecipitate. A reconstruction of a comprehensive, strictly binary, protein–protein interaction network of FRA10AC1 revealed dense interconnectivity with many disease-associated spliceosomal components and several non-spliceosomal regulatory proteins. The topological neighborhood of FRA10AC1 depicts an interactome associated with multiple severe monogenic and multifactorial neurodevelopmental diseases mainly referring to spliceosomopathies. Our results suggest that FRA10AC1 involvement in pre-mRNA processing might be strengthened by interconnecting splicing with transcription and mRNA export, and they propose the broader role(s) of FRA10AC1 in cell pathophysiology.

## 1. Introduction

Fragile sites (FSs) are genetic loci that are highly conserved across mammalian chromosome evolution [1,2], manifesting chromosomal gaps or breaks in metaphase spreads of cells grown under culture conditions that partially inhibit DNA replication or repair. Depending on their frequency in humans, FSs are distinguished as common and rare [3,4,5]. Rare FSs in human are expressed in cell cultures mainly under folate deprivation. They all carry expanded tandem trinucleotide repeats which correspond to dynamic mutations of normally occurring polymorphic (CGG)n sequences [6]. The manifestation of fragility is caused by a significant expansion of the repeat at the mutated gene allele, resulting in the hypermethylation of the neighboring CpG-rich region. A number of folate-sensitive FSs may have clinical implications [7,8,9,10]. A typical example is *FRAXA*, which is responsible for Fragile X syndrome, which is characterized by intellectual and developmental abnormalities due to either the dynamic expansion of a 5′ UTR (CGG)n repeat, or missense and nonsense mutations of the causative *FMR1* gene [11,12]. Notably, FMRP, the *FMR1*-encoded protein, is a multifunctional regulatory protein involved in neuron development and synaptic plasticity through participating in various steps of RNA metabolism and mRNA alternative splicing [13].

*FRA10A,* mapped to 10q23.3, is one of the two rare and five common FSs of human chromosome 10 [14] and has been previously detected in individuals with mild intellectual disability and developmental delay [7,10,15,16,17]. We have shown that *FRA10A* expression is caused by the expansion of a polymorphic (CGG)_8–14_ repeat localized within the 5′ UTR of *FRA10AC1*, a gene widely expressed in human tissues [18]. *FRA10AC1* has no detectable paralogs. Due to alternative splicing, it encodes five FRA10AC1 protein isoforms characterized by considerable heterogeneity towards their carboxy-terminus [18], presumably suggesting distinct, tissue-specific biological roles. The most abundant isoform is a ubiquitously expressed nuclear 315 aa polypeptide that is highly conserved in all multi-cellular [18] and some unicellular eukaryotes [19], suggesting it plays an essential role in cell physiology. In *FRA10A* carriers, the (CGG)_n_ repeat expands beyond 200 copies and becomes hypermethylated, resulting in the silencing of the respective allele and in gene haploinsufficiency [18]. By mass spectrometry analysis of spliceosomal subcomplexes that had been affinity-purified from *HeLa* nuclear extracts, FRA10AC1 was identified as a component of the B-activated, C, and post-catalytic P subcomplexes [20,21,22,23]. Furthermore, in the context of a targeted large-scale yeast two-hybrid interaction matrix screen of *HeLa* spliceosomal core and non-core proteins, FRA10AC1 was found to interact with fifteen members of the spliceosome proteome [24], suggesting that it plays a role in pre-mRNA splicing. Along the same lines, a suppressor screen, carried out on *Chlamydomonas reinhardtii* mutants manifesting very short or absent flagella due to either acceptor or donor splice site mutations, identified a frameshift mutation in the *FRA10AC1* ortholog that rescues both mutants. This indicates the possible role of *FRA10AC1* in facilitating the recognition of or interaction between the acceptor and/or donor splice site and the branch point [19]. Interestingly, reminiscent of the clinical phenotypes caused by mutants of FMRP [12,13], clinical phenotypes have been reported that are caused by the biallelic loss of function variants of *FRA10AC1*, which is characterized by strong intellectual disability and developmental delay [25,26,27]. Furthermore, in the context of a genome-wide association study (GWAS) aiming to identify genetic variants associated with amyloid-β 1–42 peptide levels in cerebrospinal fluid other than *APOE* ε4, as important biomarkers for Alzheimer’s disease, two *FRA10AC1* SNPs—one intronic (a cis-eQTL) and one located less than 1kb upstream of the CGG repeat—were the top hits [28].

This work aims to further elucidate the biological characteristics of FRA10AC1 regarding its participation in spliceosome protein complexes and its association with the related neurodevelopmental phenotypes. We confirm the direct interaction of FRA10AC1 with two spliceosomal proteins, ESS2 and SF3B2, by using a GST-pull down assay, an alternative to the yeast two-hybrid interaction matrix screen performed previously [24], and we confirm the detection of a physical interaction between the endogenous FRA10AC1 and ESS2 in *HeLa* cells, a result supporting the in vivo functional synergy of the two proteins. In contrast, the FRA10AC1-SF3B2 interaction could not be detected at the level of endogenous protein abundance. In addition, we mapped the FRA10AC1 and ESS2 binding domains. Evidence of tissue co-expression is a necessary prerequisite to confirm the presence of true protein interactions. PPI data were complemented and supported by RNA in situ hybridizations showing the specific co-expression of *FRA10C1*, *ESS2*, and *SF3B2* homologs in a number of mouse embryos and adult brain tissues. The *FRA10AC1* median expression profile clusters with that of *ZCCHC10*, *CWC27*, *ZNF830*, and *ESS2* in human brain tissues and in the spinal cord, indicating the close functional cooperation of these spliceosomal proteins. A reconstruction of the FRA10AC1 interactome suggested the putative role of this protein in interconnecting splicing with transcription mechanisms and mRNA export machinery, and also, it suggested the protein’s broader role as a partner of proteins involved in a variety of cell functions other than splicing. Finally, the topological neighborhood of FRA10AC1 associates it with a disease network of twenty clinical phenotypes, mostly referring to severe neurodevelopmental and neurodegenerative disorders. 

## 2. Material and Methods

### 2.1. Protein–Protein Interaction (PPI) Data Retrieval and Protein Network Reconstruction

FRA10AC1 PPI data were extracted from PICKLE (version 3.3), a meta-database of the direct PPI network of the human and mouse proteomes [29,30]. PICKLE integrates all publicly available primary PPI datasets in a way that allows heterogeneous networks to be reversibly normalized to different levels of genetic reference without the deprivation of the original PPI information [31,32]. In order to obtain experimentally verified, strictly binary PPIs, the “cross-checking” filtering mode was used. A visualization of the protein interactome was performed by the Cytoscape software platform (version 3.8.2, http://www.cytoscape.org/, accessed on 15 December 2011). Gene/protein biological characteristics were obtained from UniProt KB (release 2022_05) [33], OMIM (https://www.omim.org/, accessed on 1 December 2022), GWAS Catalogue (https://www.ebi.ac.uk/gwas/, accessed on 3 November 2022), and the literature.

### 2.2. GTEx Data Collection 

Co-expression profiles of the *FRA10AC1* gene and its spliceosomal first neighbors were deduced from GTEx Portal V8 (https://gtexportal.org/home/, accessed on 15 November 2022) for all available human tissues and cells using the multi-gene query [34]. The heatmap depicts TPM values and clusters the genes based on hierarchical clustering. For *ESS2*, its previous symbol, *DGCR14*, was used. 

### 2.3. HeLa Cell Line Culture and Transfection

*HeLa* cells were obtained from the American Type Culture Collection and cultured as recommended. The transient expression of various gene constructs was determined by transfection using the calcium phosphate co-precipitation method [35]. The cells were collected after 36 h in 1x PBS containing an anti-protease cocktail (Sigma) and accordingly processed for either immunoblotting, immunoprecipitation, or pull-down assays. For immunostaining, cells were grown on coverslips after obtaining 60–70% coverage.

### 2.4. Home-Made Antibodies

Rabbit polyclonal antibodies specific to FRA10AC1 and ESS2 were raised against bacterially expressed glutathione S-transferase fusion proteins containing the FRA10AC1 1-145aa and the ESS2 25-221aa polypeptides, respectively. The selection of the above polypeptides was based on the prediction of their antigenicity using an antigenic epitope predicting tool (http://imed.med.ucm.es/Tools/antigenic.pl, accessed on 5 February 2002). The production and affinity purifications of the antibodies were performed as described in [36]. Namely, anti-FRA10AC1 and anti-ESS2 antisera were incubated with the respective His-tagged polypeptides blotted on nitrocellulose stripes in order to capture the corresponding antibodies and cleaned from the anti-GST antibody. Before use, the specificity of the antibodies was tested as follows: *HeLa* cells were transiently transfected with chimeric plasmids encoding EGFP-FRA10AC1 or EGFP-ESS2. The lysates were subjected to Western blots with the affinity-purified anti-FRA10AC1 or anti-ESS2. The result was compared with that obtained by using a commercially available anti-ΕGFP antibody. In both experiments, the protein signal, obtained by anti-FRA10AC1 or anti-ESS2, which corresponds, respectively, to ΕGFP-FRA10AC1 and ΕGFP-ESS2 fusion proteins, was the same as that obtained by anti-ΕGFP (Figure S1A,B). This result convincingly suggests the specificity of the affinity-purified antibodies.

### 2.5. GST Pull-Down Assays

ESS2 and SF3B2 cDNAs corresponding to amino acids 25–472 and 154–813, respectively, were subcloned in a pcDNA3.1HisA vector and used as templates for in vitro transcription/translation by T_N_T Quick Coupled Transcription/Translation System (Promega), in the presence of 10 μCi [^35^S] methionine (spec. activity: 1000 Ci/mmol), in a reaction volume of 25 μL. The products were incubated with a full-length glutathione S-transferase (GST)-FRA10AC1 chimeric protein, produced in a *BL21* DE3 codon+ *E. coli* strain after induction for 20 h at 28 °C with 0.1 mM IPTG and after being purified with glutathione-Sepharose beads (Amersham Biosciences Inc.). The incubation was performed in a 200 μL buffer containing 50 mM Tris-HCl pH 8.0, 100 mM NaCl, 1mM EDTA, and 0.1% Nonidet P-40, with overnight rotation at 4 °C. After extensive washing, the proteins were eluted with the Laemmli SDS sample buffer and analyzed by SDS-PAGE. The gel was incubated in a fixing solution (30% methanol, 10% acetic acid, and 3% glycerol) for 30 min and in an enhancing solution (1M sodium salicylate pH 6.6) for 1 h, and then dried and exposed on X-ray film at −80 °C. For the mapping of the FRA10AC1-ESS2 interacting regions, *ESS2* cDNA fragments corresponding to amino acids 25–472, 25–222, 223–472, 294–472, 218–316, and 390–472 were subcloned in-frame to the appropriate pGEX4T GST vector and expressed in a *BL21* DE3 codon+ *E. coli* strain, in parallel with the control GST vector, after induction with 0.1 mM IPTG for 2 h at 18 °C or 30 °C. Recombinant proteins were purified on glutathione-Sepharose beads. Approximately 2 μg of each GST fusion ESS2 protein bound to beads and equilibrated in a RIPA buffer (50 mM Tris-HCl, pH 8.0, 50 mM NaCl, 0.5% Nonidet P-40, 0.1% SDS, and anti-protease cocktail (Sigma)), was incubated with ~1.5 mg of the total lysate from *HeLa* cells or with the protein extracts of the transiently transfected *HeLa* cells expressing different FRA10AC1-EGFP fragments corresponding to either amino acids 1–145, 96–253, or 254–315. The incubation was performed overnight at 4 °C with rotation, the beads were washed, and the proteins were eluted with the Laemmli SDS sample buffer. Protein samples were analyzed by Western blotting using the home-made anti-FRA10AC1 or anti-GFP (Santa Cruz) primary antibody.

### 2.6. Co-Immunoprecipitation and Immunofluorescence

Sub-confluent cultures of *HeLa* cells were collected and handled for co-immunoprecipitation and immunofluorescence as previously described [37]. For the immunoprecipitations, 1 μg of affinity-purified home-made anti-FRA10AC1 and anti-ESS2 polyclonal antibodies was used. For the immunofluorescence, FRA10AC1 was detected by direct GFP fluorescence whereas ESS2 and SF3B2 were detected by anti-Xpress (Invitrogen) and anti-SF3B2 polyclonal antibodies (kindly provided by Prof. R. Reed, Department of Cell Biology, Harvard Medical School, Boston, MA, USA) followed by secondary Alexa Fluor 546 goat anti-mouse and Alexa Fluor 633 goat anti-rabbit antibodies (Molecular Probes), respectively.

### 2.7. RNA In Situ Hybridization

RNA in situ hybridization was performed on 16 μm thick frozen sagittal and coronal cryostat sections prepared from NMRI mouse embryos and adult mouse brains, kindly offered by Prof. G. Chalepakis, Dept. of Biology, U. of Crete, Greece. Samples were fixed with 4% paraformaldehyde in PBS and cryopreserved in 30% sucrose in PBS before being embedded in OCT compound (a PolyFreeze tissue freezing medium). For the production of riboprobes, a PCR fragment corresponding to nucleotides 250–743 of mouse *Fra10ac1* cDNA (BN000292), a ~1.1 kb *Pst*I fragment derived from mouse IMAGE cDNA *Ess2* clone 3708646 and a ~0.7 kb *Stu*I-*BamH*I fragment derived from mouse IMAGE cDNA *Sf3b2* clone 3582542 were cloned in pBluescript. Digoxigenin (DIG)-11-UTP-labeled single-stranded antisense and sense RNA probes were prepared after a linearization of the plasmids with the DIG RNA Labeling Kit (Boehringer Mannheim), and the signal was detected with an anti-DIG antibody conjugated to alkaline phosphatase and NBT/BCIP substrates (Roche) in accordance with the manufacturers’ instructions. In situ hybridizations using the respective sense riboprobes produced no appreciable signal. Sections were analyzed with a Leica microscope and recorded with a Spot digital camera. Experimental animal handling was performed according to international and national bioethical rules and conformed to the bioethics regulations of the University of Crete, Greece. 

## 3. Results

### 3.1. Validating FRA10AC1 Protein Interactions with SF3B2 and ESS2 Spliceosomal Proteins 

**Figure 1:**
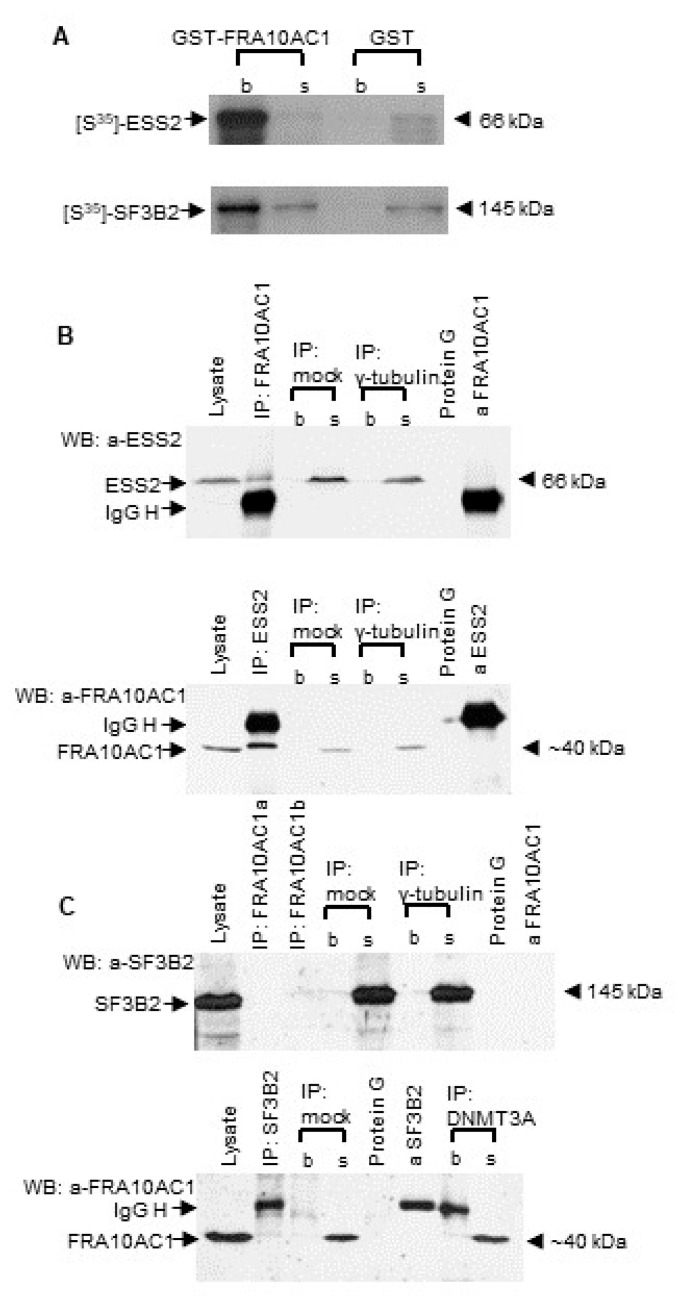
FRA10AC1 protein interaction with two spliceosomal proteins: (**A**) Autoradiogram of a GST pull-down assay of FRA10AC1 with either [S^35^]-methionine-labeled ESS2 or SF3B2. One tenth (10%) of the preparation was analyzed; b, bound; s, supernatant; GST, negative control. (**B**,**C**) Endogenous FRA10AC1 co-immunoprecipitates (co-IP) with ESS2 (**B**) but not with SF3B2 (**C**) in *HeLa* cells. The cell lysate was immunoprecipitated with (i) anti-FRA10AC1- ((**B**,**C**), upper panels), (ii) anti-ESS2- ((**B**), lower panel), and (iii) anti-SF3B2- ((**C**), lower panel) specific antibodies. For the Western blot (WB), an anti-ESS2 ((**B**), upper panel), an anti-SF3B2 ((**C**), upper panel), or an anti-FRA10AC1 ((**B**,**C**), lower panels) antibody was used. Negative controls: mock IP (IP without an antibody) and IP with anti-γ-tubulin and anti-DNMT3A antibodies. Runs of a whole protein extract (300 ug lysate), protein G-Sepharose beads, anti-FRA10AC1 ((**B**,**C**), upper panels), anti-ESS2 ((**B**), lower panel) and anti-SF3B2 ((**C**), lower panel) antibodies were also included in the analysis. IgG H, heavy chain; b, bound; s, supernatant (10% of the unbound fraction); anti-FRA10AC1a and b, 1 ug and 2 ug of the purified antibody.

Large-scale yeast two-hybrid matrix screening has suggested that FRA10AC1 interacts with several spliceosomal proteins [24]. To validate and further strengthen the information of its participation in spliceosomal multiprotein complexes, we examined the binary interaction of FRA10AC1 with two spliceosomal proteins, namely, SF3B2 and ESS2. We selected those proteins as representatives of a core and a non-core spliceosomal subunit, respectively. A way to characterize a spliceosomal protein as a core or non-core subunit has been suggested in [38]. It mainly refers to the relative abundance of the subunit following the quantitative analysis of biochemically purified spliceosomal complexes. The choice of SF3B2, a well-characterized subunit of the spliceosomal SF3b multiprotein complex, was based on the fact that it is involved in the recognition of the intron’s branch point and has an essential role in the accurate excision of introns from pre-mRNA. Thus, the confirmation of FRA10AC1-SF3B2’s in vivo interaction would place FRA10AC1 at the heart of the spliceosome. On the other hand, ESS2 (synonym: DGCR14), a non-core spliceosomal protein [24], was selected because it is the only interactor for which a functional association with FRA10AC1 has been identified. In particular, a genetic screen of *Chlamydomonas reinhardtii* has shown that ESS2 and FRA10AC1 homologs of this species suppress the same splice site mutations, suggesting their putative cooperation in splice site recognition or interaction and, overall, in splicing regulation [19]. As an alternative approach, supplementary to the yeast two-hybrid interaction, we performed a small-scale purely in vitro GST pull-down assay. As shown in Figure 1A, a full-length FRA10AC1-GST-fused protein produced in *E. coli* was able to pull-down the in vitro translated [S^35^]-methionine-labelled ESS2 or SF3B2 protein product, suggesting the direct interaction of FRA10AC1 with the two spliceosomal proteins. 

We further investigated whether the endogenous FRA10AC1 protein of *HeLa* cells is able to interact with the endogenous ESS2 and SF3B2 proteins. *HeLa* cell extracts were incubated in the presence of a home-made affinity-purified anti-FRA10AC1-specific antibody. The resulting immunoprecipitate was tested against either an anti-ESS2- or an anti-SF3B2-specific antibody, on a blot. The endogenous FRA10AC1 indeed co-precipitated with the ESS2 of the *HeLa* cells. The result was clearly confirmed by the inverse experiment in which an anti-ESS2-specific antibody was used for the *HeLa* cell extract immunoprecipitation assay (Figure 1B), thus offering strong evidence of the in vivo physical interaction between FRA10AC1 and ESS2. The corresponding test concerning the endogenous FRA10AC1 and SF3B2 protein pair failed to detect an interaction (Figure 1C), which may indicate that FRA10AC1 does not participate in the recognition of the intron branch point through the SF3b multiprotein complex. Alternatively, the binding of FRA10AC1 with SF3B2 could have been weak or prohibited under the experimental conditions used. 

### 3.2. Mapping the FRA10AC1 Protein Interacting Region

**Figure 2:**
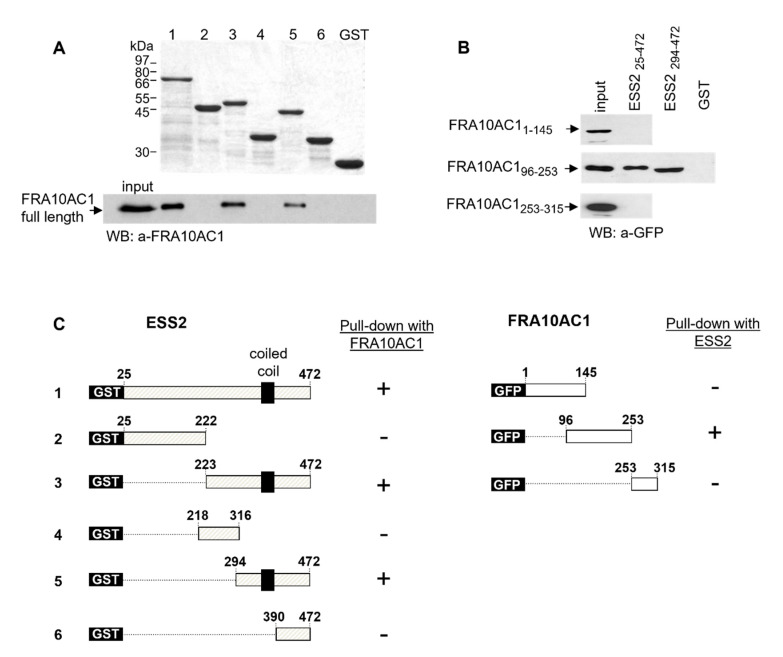
Mapping of FRA10AC1 and ESS2 interacting regions. (**A**) Determination of the ESS2 region interacting with FRA10AC1. Upper: an SDS/PAGE of a bacterially expressed series of GST-ESS2 constructs corresponding to ESS2 aa 25–472, 25–222, 223–472, 218–316, 294–472, and 390–472 (lanes 1–6, respectively), and GST alone (lane 7). Lower: a GST pull-down assay between each one of the truncated ESS2 fragments with the endogenous FRA10AC1 from *HeLa* cells (~1 mg lysate). Input represents 15% of the lysate used for the assays; an anti-FRA10C1 antibody was used for the Western blot (WB); lanes, same as in upper panel. (**B**) Determination of the FRA10AC1 region interacting with ESS2. Pull-down assay of GST- ESS2 polypeptides corresponding to ESS2 aa 25–472, 294–472, or GST alone with GFP-FRA_1–145_ (upper panel), GFP-FRA_96–253_ (middle panel), and GFP-FRA_253–315_ (lower panel) expressed in transfected *HeLa* cells. Input represents 10% of the lysate used for each pull-down assay. (**C**) Schematic representation of ESS2 and FRA10AC1 truncated polypeptides used for the mapping.

The identification of the interacting region of a protein is of considerable importance since, in most cases, proteins exert their function through interplaying with other proteins. To further characterize the FRA10AC1-ESS2’s direct interaction, we mapped the respective protein regions involved. Namely, various truncated ESS2 cDNAs fused to a GST-plasmid vector were expressed in *E. coli*, and the synthesized proteins were tested in pull-down experiments against the endogenous FRA10AC1 from *HeLa* cells. A 178 aa long polypeptide corresponding to aa 294–472 of the ESS2 carboxy-terminus was identified as the shortest region interacting with FRA10AC1 (Figure 2A). Within it, a 73 aa-long fragment spanning aa 317–390 of the protein is apparently necessary for the interaction, as deduced from the combination of all the data. Next, the GST-ESS2 (aa: 25–472) protein was used in pull-down assays against three FRA10AC1-EGFP polypeptides corresponding to aa 1–146, 96–253, and 254–315 of the FRA10AC1 produced in *HeLa* cells. As shown in Figure 2B, the central part (aa 96–253) of FRA10AC1 is sufficient for the interaction with ESS2, with the interacting region being within a 107 aa-long sequence, namely aa 146–253 of FRA10AC1.

### 3.3. Fra10ac1, Ess2, and Sf3b2 Are Co-Expressed in the Mouse Embryo and Adult Mouse Tissue

**Figure 3:**
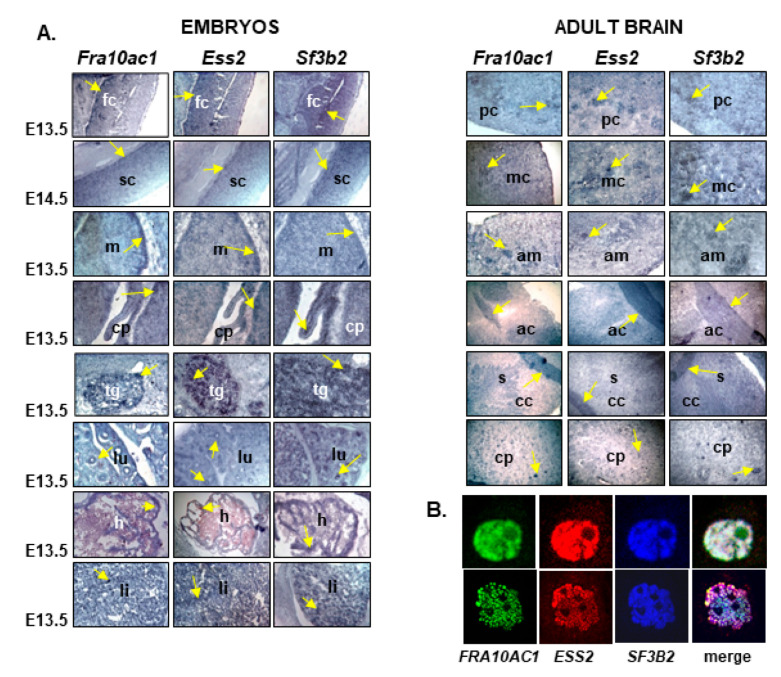
(**A**) *Fra10ac1*, *Ess2*, and *Sf3b2* show spatial co-expression in mouse tissues. Comparative expression analysis of *Fra10ac1*, *Ess2*, and *Sf3b2* through RNA in situ hybridization on sagittal mouse embryo sections and coronal sections of adult mouse brain. In embryos, co-expression of the genes was detected in frontal cortex (fc), spinal cord (sc), midbrain (m), choroid plexus (cp), trigeminal nerve (tg), lung (lu), heart (h), and liver (li). In the adult brain, overlapping regions of expression included the piriform cortex (pc), motor cortex (mc), amygdala (am), anterior commissure (ac), and caudate putamen (cp). (**B**) Co-localization of FRA10AC1, ESS2, and SF3B2 in the *HeLa* cell nucleus. FRA10AC1 was detected by direct GFP fluorescence. ESS2 and SF3B2 were detected by anti-Xpress and anti-SF3B2 antibodies followed by fluor secondary antibodies. In the majority of cells, a diffused nucleoplasmic pattern was observed (upper panel), while in ~10% of cells, a punctuate pattern was detected in over-expressing FRA10AC1 and ESS2 cells (lower panel). Nuclear staining was performed with DAPI (not shown). The merged images reveal the co-localization of the proteins.

The binary interactions between FRA10AC1, ESS2, and SF3B2 were suggested in the context of a large-scale yeast two-hybrid matrix screen [24] and validated here by complementary assays (Figure 1 and Figure 2). However, it has been noted that protein–protein interaction techniques account for a considerable number of false positives due to various inherent methodological constrains [39,40]. Furthermore, these techniques may detect “true” interactions that do not exist in vivo because the referred proteins are never expressed in the same tissue, developmental stage, and/or subcellular compartment. To further support the functional relationship of FRA10AC1, ESS2, and SF3B2, we examined the spatial expression patterns of their mouse homologs, and questioned whether these genes are co-expressed in various mouse tissues. Accordingly, we performed RNA in situ hybridizations on sagittal mouse embryos and adult coronal brain sections. All three genes, *Fra10ac1*, *Ess2*, and *Sf3b2*, were ubiquitously expressed and showed an extensive pattern of co-expression in all the tissues examined. Strong hybridization signals were observed in particular regions of the developing embryo nervous system, i.e., the frontal cortex, spinal cord, midbrain, choroid plexus, pons, and trigeminal nerve, as well as in the embryo lung, heart, and liver (Figure 3A). Similarly, a ubiquitous expression pattern was identified in the adult brain, with higher expression shown in the piriform and motor cortexes, amygdala, anterior commissure, and caudate putamen (Figure 3A).

### 3.4. FRA10AC1, ESS2, and SF3B2 Co-Localize in the HeLa Cell Nucleus

Along the same lines, we examined the subcellular localization of FRA10AC1 in comparison with that of ESS2 and SF3B2. Co-localization of all three proteins exclusively in the nucleoplasm was observed when EGFP-FRA10AC1 and Xpress-ESS2 were transiently expressed in *HeLa* cells, while the endogenous SF3B2 was detected by using an anti-SF3B2-specific polyclonal antibody (Figure 3B). 

### 3.5. Reconstruction of the FRA10AC1 Protein Interactome 

**Figure 4:**
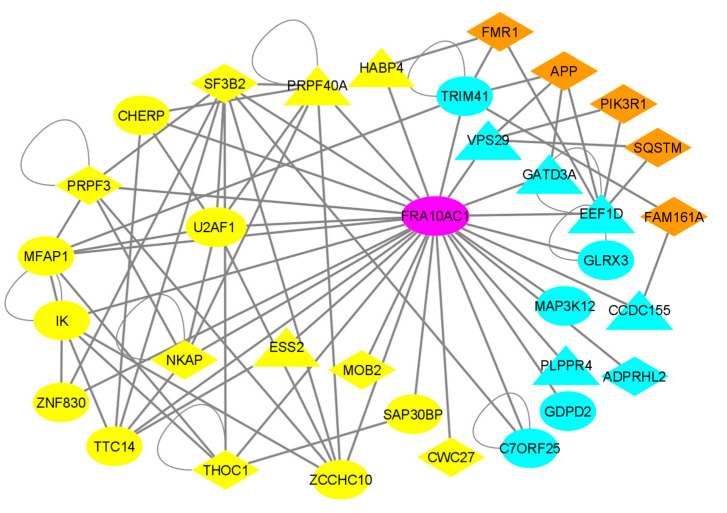
Reconstruction of the FRA10AC1 PPI network. The first neighbors of FRA10AC1 and any interactions between them are shown. “Yellow” nodes represent proteins being characterized as spliceosomal components. “Turquoise” proteins represent FRA10AC1 interactors not involved in pre-mRNA processing (non-spliceosomal). The network is enriched with five disease-causative proteins, shown in orange, interconnected with at least two “turquoise” FRA10AC1 interactors. “Diamond” and “triangle” nodes correspond, respectively, to disease-causative and disease-associated proteins.

It has been broadly declared that proteins interacting to each other may be involved in the same or similar biological functions. Therefore, knowing the functional characteristics of a protein may offer valuable insights into the function of its interacting protein(s) with unknown functions. Regarding genetic lesions, this principle supports the statement that interacting proteins may be involved in the same or closely related diseases, with the opposite being valid also [41,42,43]. In order to obtain clues regarding the overall impact of the FRA10AC1 function, we reconstructed a comprehensive protein–protein interaction (PPI) network for this protein by using PICKLE (release 3.3), a meta-database for direct human PPIs, in its cross-checked filtering mode. The FRA10AC1-reconstructed PPI network is composed of 28 first-neighbor protein nodes interconnected by 58 direct PPIs (Figure 4). All the PPIs and the experimental methods supporting each PPI are listed in Table S1. A number of FRA10AC1 interactors have been identified as spliceosomal components in proteomic studies that characterize the composition of either the whole spliceosome or of spliceosome subcomplexes of *HeLa* cells [20,21,22,23,38]. A list of known or putative function(s) of all FRA10AC1 interactors and their suggested subcellular locations and involvement in disease are listed in Table S2. Apart from TTC14 (tetratricopeptide repeat protein 14, UniProt id: Q96N46), a nucleic acid binding protein with an unknown function, biochemically identified as a spliceosomal component [20,21,23], sixteen of the FRA10AC1 interactors are implicated in pre-mRNA processing. Nine of them, namely U2AF1, SF3B2, PRPF3, MFAP1, ESS2, PRPF40A, IK, CHERP, and HABP4, have been identified as splicing factors; four, NKAP, SAP30BP, EEF1D, and ZCCHC10, are associated with transcription regulation; three, THOC1, CWC27, and MOB2, are characterized as components of the mRNA transport machinery. Interestingly, nine of these proteins are involved in a number of genetic neurological and developmental disorders, mainly spliceosomopathies [44] (see Table S2 and Table 1, and Discussion). 

In addition to the spliceosome-associated interactors, there are 11 proteins scored as FRA10AC1 binary interactors, which do not participate in pre-mRNA processing. Apart from C7orf25, a nuclear protein of an unknown function, the other 10 proteins are implicated in various functions, with several being associated with neurological diseases (see Table S2 and Table 1, and Discussion). Namely, they are associated with (i) cell signaling, i.e., MAP3K12, a component of a MAPK pathway enhancing the transcription of APP and GDPD2, a negative regulator of the urokinase receptor signaling network); (ii) the assembling of regulatory polyprotein complexes, i.e., EEF1D, a translation regulator participating in the elongation factor-1 complex, CCDC155, a component of the LINC complex required for telomere attachment to the nuclear envelope in the meiotic prophase, VPS29, which is involved in the endosomes of the trans-Golgi cargo-selective complex, and GLRX3, an assembly factor of cytosolic iron-sulfur (Fe-S cluster); (iii) enzymatic activities, i.e., those of ADPRHL2, which is involved in DNA damage and repair, with ADP-ribosylhydrolase activity, such as that of TRIM41, an essential E3 ligase involved in proteasome processes, PLPPR4, a lipid phosphate phosphatase modulating excitation at the synaptic junction, and GATD3A, a mitochondrial matrix deglycase interacting with the mitochondrial protein translation machinery. All these interactions may indicate the broader role of FRA10AC1 in a variety of cell functions other than splicing.

### 3.6. Co-Expression Analysis of FRA10AC1 Spliceosomal Interactors

**Figure 5:**
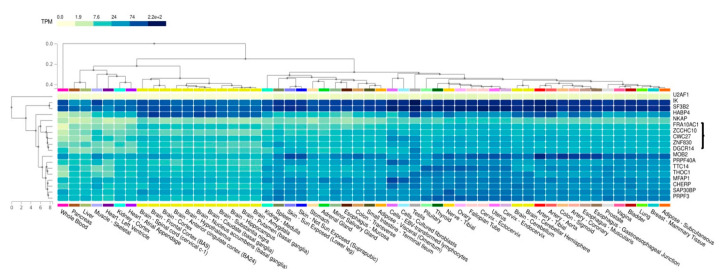
Comparative expression analysis of *FRA10AC1* with its interactors genes involved in pre-mRNA processing. The hierarchical clustering of gene transcripts based on the pattern of their median expression across tissues as provided by the GTEx algorithms is shown. *FRA10AC1* clusters with *ZCCHC10*, *CWC27*, *ZNF830*, and *ESS2* (*DGCR14*) based on their expression profiles (grouped with a brace), with *NKAP* sharing a closely related expression pattern. Various groups of tissues share similar expression patterns for these genes, e.g., all brain tissues except cerebellum, the latter showing a relatively higher expression.

It is generally accepted that genes sharing a common expression pattern are usually involved in the same or related functions. We investigated the *FRA10AC1* expression pattern comparatively to the genes encoding its spliceosomal interactors. The relative transcriptomics data obtained from the GTEx database shown in a heatmap based on hierarchical clustering are presented in Figure 5. Apart from *SF3B2* and *IK*, which are expressed at relatively higher levels, all other genes show moderate or low expression in most of the available tissues. The *FRA10AC1* expression pattern clusters with that of *ZCCHC10, CWC27, ZNF830,* and *ESS2* (*DGCR14*) in all brain tissues and the spinal cord. Notably, FRA10AC1, CWC27, ZNF830, and ESS2 have been previously identified in the same spliceosomal subcomplexes exclusively [20,21,22,23]. This observation argues in favor of the potentially close functional synergy of these spliceosomal proteins, characterized by binary interactions with FRA10AC1, during the spliceosome stepwise assembly. 

## 4. Discussion

*FRA10AC1* has been characterized as the causative gene for the expression of the folate-sensitive fragile site *FRA10A*. The expansion of a 5′UTR (CGG)n repeat leads to the hypermethylation of this region, resulting in the silencing of the *in cis* allele [18]. Until recently, no specific phenotypes were associated with the manifestation of *FRA10A* and *FRA10AC1* haploinsufficiency. Although several studies had previously referred to clinical observations associating FRA10A expression with neurological abnormalities and/or intellectual impairment [7,8,15,16,17], the question of whether these observations were due to ascertainment bias remained open. However, a recent report [25] documented the causative relationship of biallelic *FRA10AC1* pathogenic mutants with severe neurodevolopmental clinical phenotypes including craniofacial and corpus callosum abnormalities consistent with autosomal recessive inheritance (MIM No.: 620113). These interesting results were enriched quickly thereafter by similar findings associating the homozygous deletion of the 5′ part of *FRA10AC1*, and the homozygous *FRA10AC1* nonsense mutant, with developmental delay, growth retardation, and facial dysmorphism [26]. Similarly, in an another recent publication, a homozygous nonsense mutation leading to a premature stop codon in *FRA10AC1* exon 8 was linked to neurodevelopmental impairment and dysmorphic features [27]. 

Within the last fifteen years, the experimentally determined human protein interactome has been expanded significantly, currently covering more than 80% of the UniProtKB/Swiss-Prot-reviewed complete human proteome with more than 220,000 binary PPIs documented by either small-scale or high-throughput methodologies [30]. However, about 40% of these PPIs have been determined merely by a single detection method reported in a single publication [30,31]. Thus, there is a need to independently assess reported PPIs by employing a variety of experimental methods. In order to further investigate the biological characteristics of this nuclear non-core spliceosomal protein, which is well conserved from fission yeast to humans, we validated its direct interaction with a core spliceosomal protein, SF3B2, and a non-core one, ESS2, employing an in vitro pull-down small-scale assay as an alternative to the yeast two-hybrid matrix screen used by Hegele et al. [24]. In addition, co-immunoprecipitation data regarding the endogenous FRA10AC1, ESS2, and SF3B2 of HeLa cells suggested the functional cooperation of FRA10AC1 with ESS2 but not with SF3B2, showing that FRA10AC1 may not participate in the SF3b multiprotein complex involved in the recognition of the intron branch point. The validation of the endogenous FRA10AC1–ESS2 direct interaction poses the question of whether the functional cooperation of these proteins is important for splicing regulation in mammalian cells, as it has been suggested for ESS2, which fosters precise mRNA splicing in *Caenorhabditis elegans* [45] and suppresses splicing at weak splice sites in *Arabidopsis thaliana* [46]. To our knowledge, under in vitro and/or human cell culture conditions, the inhibition or loss of FRA10AC1 activity does not affect precise mRNA splicing. von Elsner et al. [25], using an in vitro splicing assay in the primary fibroblasts of patients with *FRA10AC1* mutations, investigated whether FRA10AC1 deficiency suppresses the aberrant splicing of an engineered EGFP reporter gene. However, the results obtained could not confirm the work of Lin et al. [19] on *Chlamydomonas reinhardtii*, which reported that FRA10AC1 and ESS2 homologs of this unicellular eukaryote may recognize mutated splice sites and restore accurate pre-mRNA splicing. Similarly, RNA sequencing data from cultured primary fibroblasts of patients with loss-of-function mutations of FRA10AC1 did not detect major qualitative changes in the respective mRNA splicing pattern [25,26]. Taking into consideration that FRA10AC1 structural variants affect neurons [25,26], and that neural cells are more vulnerable in splicing defects and perturbations in pre-mRNA splicing [47], FRA10AC1–ESS2 functional cooperation in splicing regulation could be tested in a neural cell background, i.e., by using neuronal cell cultures developed from induced pluripotent stem cells (iPSCs) derived from FRA10AC1-deficient patients. In any case, the significance of the functional contribution of FRA10AC1 and ESS2 in splicing may vary between the relatively simple spliceosomal machinery of a unicellular organism and the mammalian counterpart characterized by a much higher complexity. Finally, mapping the interacting domains of FRA10AC1 and ESS2 may offer a valuable information explaining their possible functional consequences due to structural FRA10AC1 or ESS2 variants with defects in their domains. This approach is consistent with the use of network analysis for genotype–phenotype correlations since mutations in the interacting protein domains may perturb or rewire the local structure of the network, leading to disease phenotypes [48].

Since FRA10AC1 has been sufficiently documented as a peripheral protein of the spliceosome, the reconstruction of a comprehensive FRA10AC1 binary PPI network showed its connectivity with 17 proteins involved in RNA processing in a well-interconnected subnetwork, supporting its functional role in this machinery (Figure 4 and Tables S1 and S2). FRA10AC1 partners belong to various spliceosomal subcomplexes and have different stoichiometries based on their relative abundance [24]. FRA10AC1 has been isolated as a component of the catalytically active spliceosomal B-activated and C complexes, as well as from the post catalytic complex P. It displays a similar temporal expression profile to that of ESS2, CWC27, and ZNF830 proteins regarding their participation in the same subcomplexes during spliceosome assembly [20,21,22,23]. Accordingly, our analysis, deduced from the GTEx resource database, revealed that *FRA10AC1* shares a closely related expression profile with *ZCCHC10*, *CWC27*, *ZNF830*, and *ESS2* (*DGCR14*) across 54 non-diseased human tissues including the brain (Figure 5). Moreover, the co-expression of the mouse *FRA10AC1*, *ESS2*, and *SF3B2* homologs in a number of embryo tissues and adult brain sections (Figure 3) indicated co-regulation and functional associations among the respective encoded proteins. In agreement with this, it has been shown that both FRA10AC1 and ESS2 orthologs of *Chlamydomonas reinhardtii* are sufficient to suppress splice site gene mutations, indicative of their participation in splice site recognition [19]. Similarly, although no functional association has been determined between CWC27 and FRA10AC1 yet, a recent study applying RNA-seq in the retina of a mouse model carrying mutant *CWC27* alleles has shown that *FRA10AC1* is among the top upregulated genes, possibly implying its ability to partially compensate this “loss-of-function” mouse genotype [49]. 

Presently, sixteen of the FRA10AC1 interactors (Figure 1) have been characterized as having a function in pre-mRNA processing. Nine of them are splicing factors, including U2AF1, which recognizes the conserved terminal AG present at the metazoan 3’ splice sites [50]; SF3B2, an essential component of the SF3b splicing factor that binds to pre-mRNA and tethers U2 snRNP to the branchpoint site [51]; PRPF3, a component of the U4/U6.U5-tri-snRNP complex [52]; MFAP1, which is required for pre-mRNA processing in *D. melanogaster* [53] and affects alternative splicing in *C. elegans* [54]; PRPF40A, the putative ortholog of the yeast U1 snRNP splicing factor Prp40 bridging the interaction of intron ends [55]; IK, CHERP, and HABP4, which affect splice site selection [56,57,58]; and ESS2, which is a non-core splicing factor, as discussed in previous paragraphs. Four proteins have been associated with transcription regulation, namely NKAP, which activates NF-kappaB [59]; SAP30BP, which is involved in transcriptional repression [60]; EEF1D, whose long isoform is implicated in the transcriptional activation of heat-shock responsive genes [61]; ZCCHC10, which is involved in the suppression of the telomerase reverse transcriptase (*hTERT*) gene transcription [62]. Finally, three interactors are associated with the mRNA export machinery, namely THOC1, a component of the THO complex [63]; CWC27, which is involved in the recruitment of eIF4A3, a core exon junction complex (EJC) subunit [64]; MOB2, a subunit of the EJC interactome [65]. Therefore, based on the biological role of the above 16 interactors, we support the hypothesis that FRA10AC1 participates in pre-mRNA processing by interconnecting splicing with transcription mechanisms and the mRNA export machinery. Of note is that the majority (9/16) of the above FRA10AC1 interactors comprise part of the spliceosomopathy spectrum of neurodevelopmental diseases (Table 1 and Figure 4).

Interestingly, 11 additional proteins complementing the FRA10AC1 interactome (Figure 4) are not involved in pre-mRNA splicing (Table S2 and references therein). Instead, they participate in a variety of regulatory processes in a range of important activities from the various steps of genetic information flow to the assembling of the meiosis machinery and the modulation of synaptic junction excitation, exerting their function through cell signaling and the formation of multi-protein regulatory complexes or enzymatic activity. Six of them are disease-associated (Table 1 and Figure 4). The interconnection of FRA10AC1 with these proteins indicates its broader role in cell physiology beyond that of its involvement in pre-mRNA processing, which nevertheless remains to be experimentally investigated in depth. 

A noteworthy dense network of disease-proteins associated with the spliceosome emerged through the reconstruction of the FRA10AC1 interactome (Figure 4). The respective gene–disease correlations are listed in Table 1. Six “spliceosomal” genes and one “non-spliceosomal” disease-causative gene, *SF3B2*, *PRPF3*, *THOC1*, *CWC27*, *NKAP*, *MOB2*, and *ADPRHL2*, are connected to severe neurological and/or developmental diseases including craniofacial microsomia, hearing loss, an X-linked intellectual developmental disorder, different types of retinitis pigmentosa, and childhood-onset neurodegeneration. In addition, three “spliceosomal” and five “non-spliceosomal” genes, *PRPF40A*, *ESS2*, *HABP4*, *EEF1D*, *CCDC155*, *VPS29*, *PLPPR4*, and *GATD3A*, have been associated with various types of neurodevelopmental and neurodegenerative phenotypes, including schizophrenia, Alzheimer’s, and Parkinson’s. Furthermore, based on PICKLE meta-database PPI data, five nodes, *FMR1*, *FAM161A*, *APP*, *SQSTM*, *PIK3R1*, which are well-connected with at least two “non-spliceosomal” FRA10AC1 interactors (Table S1), reveal the additional important correlations of the FRA10AC1 network with severe well-described neurodevelopmental and neurodegenerative diseases including Fragile X syndrome, Alzheimer’s disease, cerebral amyloid angiopathy, amyotrophic lateral sclerosis, and SHORT syndrome (Figure 4, Table 1). With FRA10AC1 being well-connected by three binary interactors, namely VPS29, GATD3A, and EEF1D, to APP (amyloid β A4 precursor protein) (Figure 4), the previously reported information characterizing two *FRA10AC1* SNPs biomarkers of Alzheimer’s disease [30] is further supported. Similarly, the topological proximity of FRA10AC1 with FMR1, with the two proteins being second neighbors interconnected by three protein nodes, HABP4, TRIM41, and EEF1D, may reveal functional correlations between FRA10AC1 and FMR1 in the manifestation of Fragile X syndrome or of the FRA10AC1-causative neurodevolopmental clinical phenotypes described recently [27,28,29]. 

Overall, these network-based valuable findings obtained in the context of spliceosomopathies support the hypothesis regarding the phenotypic impact of single-gene abnormalities that causally affect various functional processes, propagated through complex protein interconnections across a topological network neighborhood. This functional interdependency may, at least partly, explain co-morbidity phenomena at the molecular level. 

## Figures and Tables

**Table 1:** Disease-causative and disease-associated genes in FRA10AC1 interactome.

		Disease	Reference	Inheritance	Association
**Spliceosomal FRA10AC1 interactors**	***PRPF40A***	Memory dysfunction in frontotemporal lobe dementia, Male puberty timing (late vs. average onset voice breaking)	GWAS catalog		#
	***SF3B2***	Craniofacial microsomia	MIM No. 164210	AD	
	***PRPF3***	Retinitis pigmentosa 18	MIM No. 601414	AD	
	***THOC1***	Late-onset, progressive, non-syndromic hearing loss	PMID: 32776944	AD	
	***CWC27***	Retinitis pigmentosa & neurological defects	MIM No. 250410	AR	
	***ESS2***	Schizophrenia	PMID: 16432632		#
	***NKAP***	Hackmann-Di Donato-type X-linked syndromic intellectual developmental disorder	MIM No. 301039	XLR	
	***HABP4***	Cognitive ability	GWAS catalog		#
	***MOB2***	Neuronal mispositioning and periventricular nodular heterotopia (probable cause of)	PMID: 29593499	AR?	
**Non-spliceosomal FRA10AC1 interactors**	***EEF1D***	Neurodevelopmental disorder (specific to the long protein isoform)	PMID: 36344539		#
	***ADPRHL2***	Neurodegeneration, childhood-onset, stress-induced, with variable ataxia and seizures	MIM No. 618170	AR	
	***CCDC155***	Idiopathic non-obstructive azoospermia	PMID: 29790874		#
	***VPS29***	Schizophrenia, neurodegenerative diseases (Alzheimer’s, Parkinson’s, frontotemporal lobar degeneration)	PMID: 29755290, 32398722		#
	***PLPPR4***	Intellectual disability	PMID: 32388443		#
	***GATD3A***	Crohn’s disease; Parkinson’s disease	PMID: 18587394; 35307029		#
**FRA10AC1 second neighbors (selected)**	***FMR1***	Fragile X syndrome, Fragile X tremor/ataxia syndrome, Premature ovarian failure 1	MIM No. 300624; 300623; 311360	XLD, XL	
	***FAM161A***	Retinitis pigmentosa 28	MIM No. 613596	AR	
	***APP***	Alzheimer disease, familial; cerebral amyloid angiopathy	MIM No. 104300; 605714	AD	
	***SQSTM***	Amyotrophic Lateral Sclerosis 3, Paget Disease of Bone 3; Neurodegeneration with ataxia, dystonia, and gaze palsy, childhood-onset	MIM No. 616437; 167250; 617145	AD, AR	
	***PIK3R1***	SHORT Syndrome, Immunodeficiency 36	MIM No. 269880; 616005	AD	

Note: AR: autosomal recessive; AD: autosomal dominant; XLR: X-linked recessive; XLD: X-linked dominant; #: association of the corresponding FRA10AC1 interactor with the respective disease.

## Data Availability

The data presented in this study are available within this article and in the supplementary material.

## Supplementary Materials

The following supporting information can be downloaded at: https://www.mdpi.com/article/10.3390/genes14030568/s1. Figure S1: testing home-made anti-FRA10AC1 and anti-ESS2 antibody specificity; Table S1: List of direct protein interactions of FRA10AC1 with its first neighbors and any interactions between them; Table S2: First neighbors of FRA10AC1 and their characteristics.
