# Supplementary material for: Reconstruction of a Comprehensive Interactome and Experimental Data Analysis of FRA10AC1 May Provide Insights into Its Biological Role in Health and Disease
Source: Genes (Basel). 2023 Feb 24;14(3):568. doi: 10.3390/genes14030568 (PMC10048706; doi:10.3390/genes14030568)
Supplement: Supplementary file 1 [file genes-14-00568-s001.zip › Sarafidou et al_Legend Fig.S1.pdf]

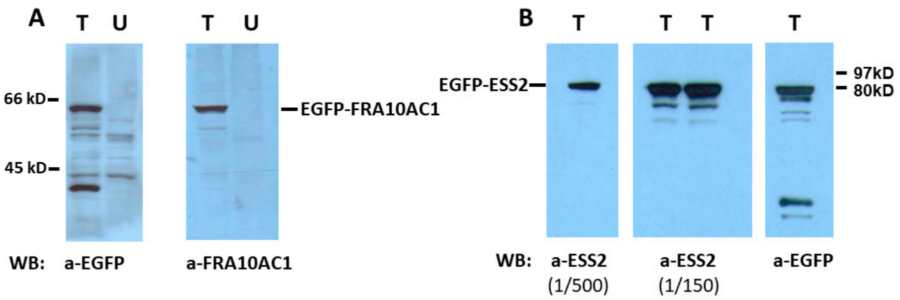

Figure. S1. Testing home-made anti-FRA10AC1 and anti-ESS2 antibody specificity. (A) *HeLa* cell lysates transfected with an EGFP-FRA10AC1 recombinant plasmid were subjected in western blot analysis using either anti-EGFP or affinity-purified anti-FRA10AC1 antibodies. (B) Similarly, *HeLa* cell lysates transfected with an EGFP-ESS2 recombinant plasmid were subjected in western blot analysis using either anti-EGFP or two different dilutions of affinity-purified anti-ESS2 antibody. Abbreviations: T, transfected cells ; U, untransfected cells; WB, western blot; numbers in kD indicate protein size markers.
